# Lipoprotein-associated Phospholipase A2 Is Associated with Risk of Mild Cognitive Impairment in Chinese Patients with Type 2 Diabetes

**DOI:** 10.1038/s41598-017-12515-z

**Published:** 2017-09-26

**Authors:** Rongrong Cai, Rong Huang, Jing Han, Haixia Sun, Jie Sun, Wenqing Xia, Sai Tian, Xue Dong, Yanjue Shen, Shaohua Wang

**Affiliations:** 1 Department of Endocrinology, The Affiliated ZhongDa Hospital of Southeast University, Nanjing, China; 2 Medical school of Southeast University, Nanjing, China; 3 Department of Endocrinology, Yixing People’s Hospital, Yixing, China; 4 Department of Endocrinology, Nanjing First Hospital, Nanjing Medical University, Nanjing, China; 5 Department of Endocrinology, Wuxi No.2 People’s Hospital, Wuxi, China

## Abstract

Type 2 diabetes mellitus (T2DM) is a low-grade chronic inflammatory diseases, which have been implicated in the pathogenesis of cognitive decline. We aim to evaluate associations between inflammatory markers and the risk of mild cognitive impairment (MCI) in T2DM. This study of 140 diabetic patients involved 71 with MCI and 69 controls. Clinical parameters, neuropsychological tests, high sensitivity C reactive protein (hsCRP), interleukin-6 (IL-6), lipoprotein-associated Phospholipase A2 (Lp-PLA2) mass and activity were measured. The results showed significantly higher plasma hsCRP, IL-6, Lp-PLA2 mass and activity in MCI group compared to controls. In T2DM with MCI, the Montreal Cognitive Assessment (MoCA) score was positively correlated with education level and high-density lipoprotein cholesterol (HDL-c), but inversely correlated with age, glycosylated hemoglobin, intima-media thickness (IMT), hsCRP, IL-6, and Lp-PLA2 mass and activity. Correlation analysis showed that both plasma Lp-PLA2 mass and activity were positively correlated with total cholesterol, low-density lipoprotein cholesterol, and IMT but negatively associated with MoCA score. Multivariable logistic regression analysis indicated higher hsCRP, Lp-PLA2 mass, Lp-PLA2 activity, and lower HDL-c to be independent risk factors increasing the possibility of MCI in T2DM. In conclusion, plasma Lp-PLA2 and hsCRP were found to be associated with the risk of MCI among T2DM patients.

## Introduction

The rapid growth of type 2 diabetes mellitus (T2DM) in the past few decades has aroused world public concern^1^. T2DM is associated with several complications that threaten human health^2^ and several studies have suggested that T2DM is an independent risk factor for mild cognitive decline (MCI) and dementia^3–5^. However, the pathogenesis of diabetes-related cognitive dysfunction is complex and involves many factors^6^. As an important characteristic of T2DM, inflammation is a potential mechanism explaining the association between T2DM and cognitive decline^7,8^.

Lipoprotein-associated phospholipase A2 (Lp-PLA2) is a circulating enzyme produced by inflammatory cells^9^. This enzyme belongs to the phospholpase A2 superfamily and may be regarded as an inflammatory marker^10^. In plasma, Lp-PLA2 hydrolyzes oxidized low-density lipoprotein (LDL) to produce inflammatory molecules such as lysophosphatidylcholine and oxidized nonesterified fatty acids^11^. Moreover, Lp-PLA2 can hydrolyze platelet-activating factor in platelets, monocytes, and macrophages^12^. It has been shown that both Lp-PLA2 mass and activity are higher in T2DM than in individuals without diabetes^13–15^ and that a high Lp-PLA2 level is associated with poor control of diabetes^16^. Moreover, previous studies have indicated that high Lp-PLA2 activity is a risk factor for dementia, independent of cardiovascular disease and inflammatory factors^17,18^, and a meta-analysis of more than 79,000 subjects showed that higher Lp-PLA2 mass or activity increases the risk of stroke, coronary heart disease, and vascular mortality which are associated with the development of Alzheimer’s disease (AD) and dementia^19^. Considering these findings together, we hypothesized that Lp-PLA2 might be of vital importance in mediating the role of chronic inflammation in the susceptibility of diabetic patients to cognitive impairment.

Another two potential markers associated with cognitive dysfunction and diabetes are high-sensitivity C-reactive protein (hsCRP) and interleukin-6 (IL-6)^20^. A longitudinal two-year clinical research observed that individuals within the highest tertile of serum CRP or IL-6 had a 24% higher risk for cognitive decline than those in the lowest tertile^21^. HsCRP is an acute-phase protein synthesized in the liver and is a sensitive marker of systemic low-grade inflammation^22^. Studies have suggested that an elevated CRP level is associated with global cognitive impairment^21^ and high risk for dementia^23,24^. In the brain of dementia patients, CRP has been found to be upregulated and deposited around amyloid plaques and small-vessel damages^25,26^. IL-6 is a pro-inflammatory cytokine that participates in inflammatory reactions and immune response and influences the growth and differentiation of cells in the central nervous system. Various studies have indicated increased peripheral IL-6 levels to be associated with cognitive impairment^27,28^. Furthermore, studies have shown that IL-6 may directly inhibit human hippocampal neurogenesis^29^.

In the current study, we aimed to explore the plasma levels of inflammatory markers (Lp-PLA2 mass and activity, CRP, and IL-6) in T2DM with and without MCI, and determine potential predictors of MCI among patients with T2DM. Such findings will help to clarify the potential mechanism of inflammation in cognitive decline susceptibility in T2DM and support the development of anti-inflammatory treatments for use in T2DM-associated MCI.

## Results

### Demographic, clinical, and cognitive characteristics of the study participants

A total of 140 T2DM patients were enrolled in this study: 71 patients with MCI and 69 patients without MCI. The demographic, clinical, and cognitive characteristics of the included patients are presented in Table 1. The MCI and non-MCI groups were well matched in age and gender. There were no significant differences between the two groups in history of smoking, diabetes durations, education levels, glycosylated hemoglobin (HbA1c), fasting blood glucose (FBG), triglyceride (TG), and low-density lipoprotein cholesterol (LDL-c) (false discovery rate[FDR] > 0.05). Compared with the controls, the T2DM patients with MCI had higher total cholesterol (TC) levels, intima-media thickness (IMT), and the percentage of plaque formation, whereas the high-density lipoprotein cholesterol (HDL-c) level was lower in the diabetic patients with MCI (*P* < 0.05, FDR < 0.05). The neuropsychological test scores of the MCI group were significantly lower than those of the non-MCI group (*P* < 0.001, FDR < 0.001). Plasma hsCRP, IL-6, Lp-PLA2 mass and activity were significantly higher in the MCI group (*P* < 0.05, FDR < 0.05). There were no significant differences in drug use between the two groups (Table 2).

**Table 1:** Demographic, clinical and cognitive characteristics.

Characteristic	MCI group(n = 71)	Control group(n = 69)	P value	FDR
Age (years)	60.80 ± 8.34	60.33 ± 7.90	0.733	0.831
Female, n (%)	35(49.30)	34(49.28)	0.998	0.998
Diabetes duration (years)	12.36 ± 6.60	10.31 ± 4.90	0.039*	0.060
Education Levels (years)	8.90 ± 3.27	10.12 ± 3.47	0.035*	0.060
Smoking, n (%)	27(38.03)	24(34.78)	0.690	0.838
HbA1c (%)	9.42 ± 2.19	8.66 ± 2.07	0.041*	0.058
FBG (mmol/L)	7.94 ± 2.65	7.79 ± 2.72	0.742	0.788
TG (mmol/L)	2.52 ± 1.38	2.02 ± 1.32	0.031*	0.059
TC (mmol/L)	5.64 ± 1.56	5.02 ± 1.27	0.011*	0.023*
LDL-c (mmol/L)	3.19 ± 0.83	2.99 ± 0.82	0.157	0.205
HDL-c (mmol/L)	1.14 ± 0.28	1.33 ± 0.27	<0.001*	<0.001*
IMT(mm)	1.10(0.80–1.40)	0.80(0.70–1.25)	0.002*	0.007*
Plaque formation, n (%)	52 (73.24)	34(49.28)	0.004*	0.010*
hsCRP (mg/L)	4.24 ± 2.23	2.83 ± 1.86	<0.001*	<0.001*
IL-6 (pg/mL)	3.81 ± 2.47	2.59 ± 2.01	0.002*	0.006*
Lp-PLA2 mass (ng/ml)	302.26 (247.33–448.79)	245.46 (171.81–339.27)	<0.001*	0.001*
Lp-PLA2 activity (nmol/min/ml)	32.10 ± 5.67	24.97 ± 4.80	<0.001*	<0.001*
Cognition test levels				
MoCA	21.00(16.00–23.00)	27.00(26.00–28.00)	<0.001*	<0.001*
DST	11.00(8.00–12.00)	12.00(11.00–14.00)	<0.001*	<0.001*
VFT	15.82 ± 4.20	18.72 ± 3.84	<0.001*	<0.001*
CDT	3.00(2.00–4.00)	4.00(4.00–4.00)	<0.001*	<0.001*
ST	6.15 ± 2.82	9.55 ± 2.38	<0.001*	<0.001*
TMT-A	86.00(64.00–118.00)	62.00(48.50–78.50)	<0.001*	<0.001*
TMT-B	195.50(151.50–294.75)	147.00(113.00–180.50)	<0.001*	<0.001*

*Significance, P < 0.05. Data are presented as n (%), mean ± SD, or median (interquartile range) as appropriate. Student’s t test was used to compare normally distributed quantitative variables between the MCI group and control group. The Mann-Whitney U test was used to compare asymmetrically distributed quantitative variables between the MCI group and control group. The χ^2^ test was used to compare qualitative variables between the MCI group and control group. Abbreviations: MCI, mild cognitive impairment; FDR, false discovery rate; HbA1c, glycosylated hemoglobin; FBG, fasting blood-glucose; TG, triglyceride; TC, total cholesterol; LDL-c, low-density lipoprotein cholesterol; HDL-c, high-density lipoprotein cholesterol; IMT, intima-media thickness; hsCRP, high sensitivity C reactive protein; IL-6, interleukin-6; Lp-PLA2, lipoprotein-associated phospholipase A2; MoCA, Montreal Cognitive Assessment; DST, Digit Span Test; VFT, Verbal Fluency Test; CDT, Clock Drawing Test; ST, Similarities test; TMT-A, Trail Making Test-A; TMT-B, Trail Making Test-B.

**Table 2:** Comparison of drug use between the two groups.

	Type 2 diabetes with MCI	Type 2 diabetes without MCI	P value	FDR
Number of patients	71	69		
The use of insulin (%)	47(66.20)	41(59.42)	0.407	0.930
Oral hypoglycemic drugs (%)	43(60.56)	48(69.57)	0.264	1
Biguanides (%)	34(47.89)	40(57.97)	0.232	1
a-Glucosidase inhibitor (%)	33(46.48)	36(52.17)	0.500	1
Sulfonylureas (%)	12(16.90)	14(20.29)	0.606	0.808
Nateglinide or repaglinide (%)	10(14.08)	11(15.94)	0.758	0.809
Thiazolidinediones (%)	5(7.04)	4(5.80)	1.000	1
Antihypertensive medications (%)	46(64.79)	39(56.52)	0.317	1
Angiotensin-converting enzyme inhibitors (%)	15(21.13)	10(14.49)	0.306	1
Angiotensin II receptor blockers (%)	19(26.76)	22(31.88)	0.505	0.898
b-Blockers (%)	10(14.08)	12(17.39)	0.591	0.860
Calcium channel blockers (%)	22(31.00)	19(27.54)	0.654	0.805
Diuretics (%)	13(18.31)	11(15.94)	0.710	0.811
a1-Blockers (%)	4(5.63)	1(1.45)	0.366	1
Antiplatelet medications (%)	41(57.75)	35(50.72)	0.404	1
Lipid-lowering medications (%)	45(63.38)	40(57.97)	0.512	0.819

The χ^2^ test was used to compare qualitative variables between the MCI group and control group. Abbreviations: MCI, mild cognitive impairment; FDR, false discovery rate.

### Correlations between MoCA scores and other clinical parameters

Table 3 shows the correlations between Montreal Cognitive Assessment (MoCA) scores and clinical indicators in the T2DM patients with MCI. The MoCA score was positively correlated with education level (r = 0.329, *P* = 0.005) and HDL-c (r = 0.320, *P* = 0.007) and inversely correlated with age (r = −0.290, *P* = 0.014), HbA1c (r = −0.276, *P* = 0.023), IMT (r = −0.396, *P* = 0.001), hsCRP (r = −0.327, *P* = 0.005), IL-6 (r = −0.282, *P* = 0.017), Lp-PLA2 mass (r = −0.612, *P* < 0.001), and Lp-PLA2 activity (r = −0.263, *P* = 0.027). No associations between MoCA score and diabetes duration, FBG, TG, TC, or LDL-c were observed.

**Table 3:** Relationships of the MoCA score with other clinical indicators in individuals with type 2 diabetes and MCI.

	MoCA score	
	r	P value
Age	−0.290	0.014*
Education Levels	0.329	0.005*
HbA1c	−0.276	0.023*
HDL–c	0.320	0.007*
IMT	−0.396	0.001*
hsCRP	−0.327	0.005*
IL-6	−0.282	0.017*
Lp–PLA2 mass	−0.612	<0.001*
Lp–PLA2 activity	−0.263	0.027*

*Significance, P < 0.05. Spearman rank correlation analysis was used for factors influencing the MoCA score. Abbreviations: MoCA, Montreal Cognitive Assessment; MCI, mild cognitive impairment; HbA1c, glycosylated hemoglobin; HDL-c, high-density lipoprotein cholesterol; IMT, carotid intima-media thickness; hsCRP, high sensitivity C reactive protein; IL-6, interleukin-6; Lp-PLA2, lipoprotein-associated phospholipase A2.

### Correlations between Lp-PLA2 mass, Lp-PLA2 activity and other parameters

Pearson correlation analysis for normally distributed variables and Spearman rank correlation analysis for nonnormally distributed variables were performed to investigate associations between plasma Lp-PLA2 mass, Lp-PLA2 activity and other variables (Table 4). The results suggested plasma Lp-PLA2 mass to be positively associated with TC (r = 0.187, *P* = 0.027) LDL-c (r = 0.215, *P* = 0.011), TG (r = 0.184, *P* = 0.030), and IMT (r = 0.353, *P* < 0.001) but negatively associated with MoCA score (r = −0.625, *P* < 0.001). No significant correlations were found between FBG, HbA1c, HDL-c, hsCRP, or IL-6 and Lp-PLA2 level. Lp-PLA2 activity was positively associated with TC (r = 0.182, *P* = 0.031), LDL-c (r = 0.187, *P* = 0.027), and IMT (r = 0.181, *P* = 0.032) but negatively correlated with MoCA score (r = −0.548, *P* < 0.001). There were no significant correlations of Lp-PLA2 activity with FBG, HbA1c, TG, HDL-c, hsCRP, or IL-6 (*P* > 0.05 for all).

**Table 4:** Relationship of Lp-PLA2 with other clinical indicators in all patients with type 2 diabetes.

	Lp-PLA2 mass		Lp-PLA2 activity	
	r	P-value	r	P value
FBG	0.016	0.850	−0.052	0.543
HbA1c	0.076	0.379	−0.008	0.926
TC	0.187	0.027*	0.182	0.031*
LDL-c	0.215	0.011*	0.187	0.027*
TG	0.184	0.030*	0.131	0.123
HDL	−0.153	0.071	−0.166	0.051
IMT	0.353	<0.001*	0.181	0.032*
hsCRP	0.063	0.457	0.138	0.105
IL-6	0.071	0.406	0.106	0.214
MoCA	−0.625	<0.001*	−0.548	<0.001*

*Significance, P < 0.05. Pearson or Spearman rank correlation was used to assess for relationships between Lp-PLA2 and other clinical indicators. Abbreviations: Lp-PLA2, lipoprotein-associated phospholipase A2; FBG, fasting blood-glucose; HbA1c, glycosylated hemoglobin; TC, total cholesterol; LDL-c, low-density lipoprotein cholesterol; TG, triglyceride; HDL-c, high-density lipoprotein cholesterol; IMT, intima-media thickness; hsCRP, high sensitivity C reactive protein; IL-6, interleukin-6; MoCA, Montreal Cognitive Assessment.

### Logistic regression analysis

We first conducted univariate logistic regression analysis to select independent factors that increase the selection risk of MCI in T2DM and then used multivariable regression to investigate predictors associated with the presence of MCI. All variables in Table 1 were entered in the univariate logistic regression model at step one. The results showed that T2DM patients with higher TC, IMT, hsCRP, IL-6 levels, Lp-PLA2 mass, Lp-PLA2 activity, percentage of carotid plaque, lower HDL-c levels were more likely to have MCI (Table 5).

**Table 5:** Assessment results for the risk of MCI in a simple logistic regression model in type 2 diabetes.

	β	SE of β	P-value	OR	95% CI	FDR
Age (years)	−0.007	0.021	0.731	0.993	0.953–1.034	0.828
Female, n (%)	−0.001	0.338	0.998	0.999	0.515–1.938	0.998
Diabetes duration (years)	−0.061	0.030	0.042*	0.941	0.887–0.998	0.065
Education Levels (years)	0.108	0.052	0.037*	1.114	1.006–1.234	0.070
Smoking, n (%)	−0.140	0.352	0.690	0.869	0.436–1.731	0.838
HbA1c (%)	−0.167	0.082	0.043*	0.846	0.720–0.995	0.061
FBG (mmol/L)	−0.021	0.064	0.740	0.979	0.864–1.110	0.786
TG (mmol/L)	−0.303	0.146	0.039*	0.739	0.555–0.984	0.066
TC (mmol/L)	−0.328	0.133	0.014*	0.720	0.555–0.936	0.030*
LDL-c (mmol/L)	−0.297	0.211	0.159	0.743	0.491–1.124	0.208
HDL-c (mmol/L)	2.757	0.737	<0.001*	15.748	3.714–66.779	0.001*
IMT(mm)	−1.127	0.390	0.004*	0.324	0.151–0.695	0.011*
Plaque formation, n (%)	−1.036	0.360	0.004*	0.355	0.175–0.719	0.010*
hsCRP (mg/L)	−0.394	0.111	<0.001*	0.674	0.543–0.838	0.002*
IL-6 (pg/mL)	−0.257	0.086	0.003*	0.774	0.653–0.916	0.010*
Lp-PLA2 mass (ng/ml)	−0.007	0.002	<0.001*	0.993	0.990–0.997	0.001*
Lp-PLA2 activity (nmol/min/ml)	−0.231	0.039	<0.001*	0.794	0.735–0.858	<0.001*

*Significance, P < 0.05. Simple logistic regression analysis was used to investigate the potential risk factors affecting cognitive function. Abbreviations: MCI, mild cognitive impairment; FDR, false discovery rate; HbA1c, glycosylated hemoglobin; FBG, fasting blood-glucose; TG, triglyceride; TC, total cholesterol; LDL-c, low density lipoprotein cholesterol; HDL-c, high density lipoprotein cholesterol; IMT, intima-media thickness; hsCRP, high sensitivity C reactive protein; IL-6, interleukin-6; Lp-PLA2, lipoprotein-associated phospholipase A2.

In forward stepwise multivariable regression analysis, MCI was used as the dependent variable, whereas TC, HDL-c, IMT, hsCRP, IL-6, Lp-PLA2 mass, Lp-PLA2 activity, and percentage of carotid plaque were entered as independent variables. The results showed that higher hsCRP (*P* = 0.002, FDR = 0.004), Lp-PLA2 mass (*P* = 0.016, FDR = 0.016), and Lp-PLA2 activity (*P* < 0.001, FDR < 0.001) and lower HDL-c (*P* = 0.012, FDR = 0.016) contributed to the development of MCI in T2DM (Table 6).

**Table 6:** Assessment results for the risk of MCI in a multivariable logistic regression model in type 2 diabetes.

	β	SE of β	P-value	OR	95% CI	FDR
HDL-c (mmol/L)	2.203	0.875	0.012*	9.051	1.628–50.315	0.016*
Lp-PLA2 mass (ng/ml)	−0.566	0.235	0.016*	0.568	0.359–0.899	0.016*
Lp-PLA2 activity (nmol/min/ml)	−0.247	0.049	<0.001*	0.781	0.709–0.861	<0.001*
hsCRP (mg/L)	−0.406	0.131	0.002*	0.666	0.516–0.861	0.004*

*Significance, P < 0.05. Multivariable logistic regression analysis was used to investigate potential risk factors affecting cognitive function. Abbreviations: MCI, mild cognitive impairment; FDR, false discovery rate; HDL-c, high density lipoprotein cholesterol; Lp-PLA2, lipoprotein-associated phospholipase A2; hsCRP, high sensitivity C reactive protein.

## Discussion

The major finding of this study is that the investigated diabetic patients with MCI had higher plasma hsCRP, IL-6, Lp-PLA2 mass and Lp-PLA2 activity than those with healthy cognition. MoCA score was inversely correlated with age, HbA1c, IMT, hsCRP, IL-6, Lp-PLA2 mass, and Lp-PLA2 activity and positively correlated with education level and plasma HDL-c. The results also indicated that plasma Lp-PLA2 mass and activity were positively associated with TC, LDL-c, and IMT and negatively associated with MoCA score. In addition, multivariable logistic regression analysis further showed higher hsCRP, Lp-PLA2 mass, and Lp-PLA2 activity and lower HDL-c to be independent risk factors for MCI in T2DM.

Numerous studies have indicated the association of diabetes with increased risks for dementia, MCI, and cognitive decline^30–32^. Moreover, people with diabetes in their midlife have a threefold increased risk for developing dementia in 30 years^33^. MCI is characterized by memory deficiency without loss in daily activity functions^34^. Diabetes increases the risk for MCI by 40% in both amnestic and non-amnestic^35^. Furthermore, people on anti-diabetic medications have reduced cognitive impairment^36,37^. Neuroimaging and neuropathological studies have shown that the brain atrophy rate in T2DM is three times faster than that in normal aging individuals^38,39^. Although not fully understood, glucose metabolism, insulin signaling, impairment of amyloid clearance capacity, hypercholesterolemia, vascular defect, oxidative stress, and chronic inflammation may account for the association between T2DM and cognitive impairment^40^. In this study, we mainly investigate the general effect of inflammatory marker**s** on cognitive impairment seen in the T2DM associated MCI patients.

The association of T2DM-associated MCI with the established inflammatory cytokines (Lp-PLA2, hsCRP, and IL-6) identified in this study proposes a novel assumption for its implication in inflammation-related pathogenesis. Results showed that all inflammatory biomarkers are significantly increased in the MCI group and are inversely associated with MoCA scores. Except for plasma IL-6, all cytokines remained significantly associated with MCI in T2DM after correction for multiple testing in multivariable logistic regression analysis. These results suggest a fundamental role of inflammation in the development of cognitive decline. Moreover, recent studies have implicated inflammatory markers in cognitive dysfunction. The Rotterdam Study^17^ suggested that individuals within the highest quartile of Lp-PLA2 activity exhibit a 70% higher risk for developing dementia than those in the lowest quartile^17^. The Honolulu-Asia Aging Study found that men in the upper three quartiles of hsCRP had a 3-fold increased risk for dementia irrespective of vascular risk factors^23^. Other studies have indicated that elevated hsCRP levels are associated with and poor memory^41^ and non-memory-related performance^42^. Additionally, IL-6 is also associated with the development of cognitive decline^43,44^. However, as found in other studies^45–47^, neither serum hsCRP nor IL-6 is correlated with serum Lp-PLA2 mass or activity levels in this study. This finding may be explained by the disparate inflammation pathways of the three inflammatory cytokines. Studies have suggested that Lp-PLA2 is involved in the process of inflammation arteriosclerosis^46^. Thus, Lp-PLA2 might be a highly specific marker for vascular inflammation. HsCRP is primarily activated by other cytokines in the liver and IL-6 is elevated under the circumstance of insulin resistance and visceral obesity^46^. Accordingly, hsCRP and IL-6 are mainly systemic inflammatory markers. Our study revealed that T2DM patients with MCI suffer from increased IMT and prevalence of plaque formation related to atherosclerosis. Inflammatory biomarkers Lp-PLA2, hsCRP, and IL-6 have been reported associated with atherosclerosis^48–50^, which is a risk factor for vascular disease. Epidemiologic and experimental studies have shown that both atherosclerosis and vascular disease are associated with cognitive impairments^74,75^. What’s more, it is suggested that treating vascular risk factors is important to prevent cognitive impairment^51^. Thus, it can be hypothesized that inflammatory biomarkers may collectively contribute to the vascular pathology and result in cognitive impairment.

Several studies have found potential links between inflammatory biomarkers and cognitive dysfunction, but the mechanisms remain unclear. In T2DM, Lp-PLA2 activates upstream inflammatory pathways and induces insulin resistance (IR)^52,53^. Consequently, IR leads to glucose, fat, and protein metabolism disorders related to chronic inflammation^6^. Furthermore, increased levels of inflammatory cytokines stimulate macrophages to express increased Lp-PLA2^9,54–58^, resulting in a vicious cycle. Regarding the generative process of Lp-PLA2, the correlations of LP-PLA2 mass and LP-PLA2 activity with diabetes-associated MCI may be attributable to the accumulation of inflammatory cells during inflammatory processes. Increased Lp-PLA2 can hydrolyze oxidized phospholipids to form lysophosphatidylcholine and oxidized fatty acids (primary arachidonic acid [AA]), which are inflammatory molecules^11^. In neurons, overproduction of AA can trigger depolarization of neuronal cells through calcium-dependent apoptosis^59^. AA can also be transferred to prostaglandins, which are inflammation mediators that participate in the etiopathogenesis of neurodegenerative diseases^60,61^. Additionally, hydrolysis of Lp-PLA2 can promote expression of tumor necrosis factor (TNF-α), which is a key cytokine affecting hippocampal neuroplasticity^62^. Moreover, Lp-PLA2 is an important contributor to vascular deficit and diabetes itself is a vascular risk factor^63^. Thus, the elevated Lp-PLA2 level in T2DM may be linked to cognitive decline through the vascular pathology. The specific mechanism that links hsCRP, IL-6, and cognitive deficit is not fully understood. Through activation of the complement system, CRP might contribute to immune cascade reactions that result to neurodegeneration^64^. In addition, CRP may increase the expression of adhesion molecules in vascular endothelial cells, including endothelial cells in the brain^65^. In the central nervous system, IL-6 mediates immune response and inflammatory reactions and its overexpression is harmful for cell growth^66^.

The current study revealed that plasma Lp-PLA2 mass and activity are positively associated with LDL-c and TC levels. However, in multivariable logistic regression, LDL-c and TC were not risk factors of MCI in T2DM. These results suggested that the association between Lp-PLA2 and MCI was independent of lipid levels to some extent. In this study, we also identified an association of HDL-c with MCI in T2DM. HDL-c can remove excess cholesterol from subendothelian space of cerebral microvessels^67^. Moreover, low HDL-c level is known as a risk factor of atherosclerotic diseases^68^, leading to ischemic lesions in the brain and causing cognitive decline^69^. Notably, MoCA score was positively correlated with education level in this study. It has also been suggested that higher education could increase neocortical synaptic density and delay the progression of dementia^70^. These are in line with our finding showed that educational attainment is beneficial for cognitive function. On the basis of these findings, we encourage the elderly to exercise their brain, remain active, and participate in more intellectual activities to help delay cognitive impairment.

Several limitations should be noted in the interpretation of our results. First, the results of this case-control study may be misinterpreted because of the influence of farraginous factors, random and systematic recall errors, and selection bias. For instance, the population in this study had uncontrolled diabetes; consequently, the results cannot be generalized to patients with optimal glycemic control. Second, the small sample size and single ethnicity of the surveyed subjects may limit the application of our results to other ethnic groups. Finally, the conclusion of this study should be interpreted carefully as T2DM itself may increase the risk of MCI via different mechanisms. Consequently, the relationship between Lp-PLA2 and MCI in this study may have been confounded by T2DM.

In conclusion, our study indicates that increased levels of Lp-PLA2 mass, Lp-PLA2 activity, and hsCRP are associated with MCI in T2DM patients. These biomarkers are related to inflammation, thereby suggesting potential inflammatory reaction mechanisms underlying T2DM-associated MCI. These findings suggest the need for future investigation of the role of inflammatory cytokines in the etiology of cognitive impairment in T2DM. Thus, in view of the wide availability, safety, and convenience for monitoring plasma hsCRP and Lp-PLA2 mass and activity, both of these markers should be considered for early detection of MCI.

## Methods

### Ethics

The protocol and informed consent documents were approved by the Research Ethics Committee of the Affiliated Zhongda Hospital of Southeast University. All methods in this study were performed in accordance with approved guidelines and regulations. The study protocol was explained to the participants, and all patients provided written informed consent.

### Study subjects

We performed a case-control study at the Endocrinology Division of the Affiliated Zhongda Hospital of Southeast University. We approached T2DM patients who satisfied the 1999 World Health Organization Criteria^71^ and were aged 40–80 years with a history of diabetes >3 years. We excluded subjects with the following: suffering from hypoglycemia within 3 days of neuropsychological tests and those with hypoglycemic coma, diabetic ketoacidosis, lactic acidosis or hyperosmolar nonketotic diabetic coma; cerebrovascular accidents confirmed by neuroimaging scans; a history of known neurological degenerative diseases, such as AD and Parkinson’s disease; a history of depression, severe visual or hearing loss; a history of drug or alcohol abuse within 2 months of the study; a history of taking anti-Parkinson drugs, benzodiazepines, barbiturates, short-acting anxiolytic or sedative drugs, drugs with significant anticholinergic or antimuscarinic adverse reactions, and antiepileptics within the previous 3 months; major medical illnesses (e.g., cancer, anemia, or serious infection); and thyroid disease. Participants were evaluated using 2006 European Alzheimer’s Disease Consortium criteria^72^ to assess cognitive status (normal cognition or MCI). Among the diabetic patients recruited, we identified 71 cases of MCI and 69 age-matched controls with healthy cognition.

### Clinical parameter collection and carotid intima-media thickness determination

The following patient characteristics were collected: age, gender, level of education, medication history, smoking status, and diabetes duration. Physical characteristics were measured. FBG, HbA1c, TG, TC, LDL-c, and HDL-c levels were determined from blood samples. We used color Doppler ultrasound to measure IMT, which is the distance between the luminal-intimal interface and the medial-adventitial interface. Measurements were taken at the thickest site, at two further upstream sites, and at a site located 1 cm downstream of the left and right carotid arteries. Each site was measured six times, and the average value was calculated.

### Neuropsychological tests

Neuropsychological tests, such as the MoCA, Trial Making Test-A, Trial Making Test-B, Clock Drawing Test, Verbal Fluency Test, Digit Span Test, and Word Similarity Test were conducted to evaluate cognitive function, including semantic memory, episodic memory, executive function, psychomotor speed, attention, and visuospatial skill. The Activities of Daily Living Scale, Clinical Dementia Rating Scale, Hamilton Depression Rating Scale, and Hachinski Ischemic Scale were also administered. The MoCA score was selected as the variable to evaluate the relationship between cognitive and clinical indicators based on the ability of this score to assess multiple cognitive domains. All neuropsychological tests were conducted by a skilled neuropsychiatrist from the Department of Neurology, Affiliated Zhongda Hospital of Southeast University.

### Measurement of Lp-PLA2, hsCRP, and IL-6

Blood samples (2 mL) were collected into anticoagulant-free tubes and centrifuged at 1000 × *g* for 15 min. The plasma was then stored at −80 °C until analysis. Plasma Lp-PLA2 mass was measured using an enzyme-linked immunosorbent assay (ELISA) kit (R&D Systems, Minneapolis, MN, USA), and the Cayman colorimetric assay kit was used to measure plasma LP-PLA2 activity. hsCRP and IL-6 levels were determined by high sensitivity ELISA kits (R&D Systems, Minneapolis, MN, USA). To minimize assay variance, plasma Lp-PLA2 mass and Lp-PLA2 activity were measured on the same day for all individuals.

### Statistical analyses

Data are presented as either the mean ± standard deviation (SD), median, or percentage. Normally distributed data were analyzed using Student’s *t* test and analysis of variance (ANOVA); asymmetrically distributed quantitative variables were analyzed by the nonparametric Mann-Whitney U and Kruskal-Wallis tests. The chi-squared test was used to compare qualitative variables. Relationships between MoCA scores and other parameters were analyzed by Pearson (normally distributed variables) or Spearman rank (non-normally distributed variables) correlation. Correlations between plasma Lp-PLA2 mass, Lp-PLA2 activity and other variables were also analyzed by Pearson or Spearman rank correlation. A simple logistic regression model was used to select so-called independent factors increasing the selection risk of MCI in T2DM. Forward stepwise multivariable regression analysis was then used to explore the “strongest” factors affecting the presence of MCI. We used the Benjamini- Hochberg (BH) method^73^ which control for false discovery rate (FDR) to adjust for multiple comparisons. A significant value of 0.05 was considered statistically significant.
